# Transcriptomic and metabolomic profiling reveals the drought tolerance mechanism of *Illicium difengpi* (Schisandraceae)

**DOI:** 10.3389/fpls.2023.1284135

**Published:** 2024-01-08

**Authors:** Xiu-Jiao Zhang, Chao Wu, Bao-Yu Liu, Hui-Ling Liang, Man-Lian Wang, Hong Li

**Affiliations:** Guangxi Key Laboratory of Plant Functional Phytochemicals and Sustainable Utilization, Guangxi Institute of Botany, Guangxi Zhuang Autonomous Region and Chinese Academy of Sciences, Guilin, China

**Keywords:** *Illicium difengpi*, drought stress, rehydration, transcriptome, metabolome

## Abstract

*Illicium difengpi* (Schisandraceae), an endangered medicinal plant endemic to karst areas, is highly tolerant to drought and thus can be used as an ideal material for investigating adaptive mechanism to drought stress. The understanding of the drought tolerance of *I. difengpi*, especially at the molecular level, is lacking. In the present study, we aimed to clarify the molecular mechanism underlying drought tolerance in endemic *I. difengpi* plant in karst regions. The response characteristics of transcripts and changes in metabolite abundance of *I. difengpi* subjected to drought and rehydration were analyzed, the genes and key metabolites responsive to drought and rehydration were screened, and some important biosynthetic and secondary metabolic pathways were identified. A total of 231,784 genes and 632 metabolites were obtained from transcriptome and metabolome analyses, and most of the physiological metabolism in drought-treated *I. difengpi* plants recovered after rehydration. There were more upregulated genes than downregulated genes under drought and rehydration treatments, and rehydration treatment induced stable expression of 65.25% of genes, indicating that rehydration alleviated drought stress to some extent. Drought and rehydration treatment generated flavonoids, phenolic acids, flavonols, amino acids and their derivatives, as well as metabolites such as saccharides and alcohols in the leaves of *I. difengpi* plants, which alleviated the injury caused by excessive reactive oxygen species. The integration of transcriptome and metabolome analyses showed that, under drought stress, *I. difengpi* increased glutathione, flavonoids, polyamines, soluble sugars and amino acids, contributing to cell osmotic potential and antioxidant activity. The results show that the high drought tolerance and recovery after rehydration are the reasons for the normal growth of *I. difengpi* in karst mountain areas.

## 1 Introduction

Drought events are frequent worldwide. It is predicted that extreme drought episodes will become more frequent against the background of global climate change (Dai, 2013). Being sessile, plants are extremely vulnerable to drought stress, which can lead to impaired growth and even death, resulting in enormous yield losses (Bai et al., 2006). Drought stress is thus one of the most serious environmental factors that restricts plant species distribution and diversity and crop productivity (Yang et al., 2020). The southwest karst region of China is the largest and most intensively developed ecologically fragile area among the three largest concentrated karst areas in the world, with a total area of over 500,000 km^2^ (Zhang et al., 2012). Due to slow soil formation, shallow soil layers, low organic matter content, and poor soil conservation capabilities, this region experiences geological drought. Even with enough rainfall, after several days of high temperatures and sunny weather, the soil water content decreases, and temporary drought events occur on the surface (Zhao et al., 2022). Drought disasters are the most prominent environmental factors that affect plant growth in karst regions. Short periods of extreme drought are more prone to occur in karst environments, which are characterized by periodic water deficiency. The recovery ability after rehydration is important for the successful adaptation of plants in karst ecosystems prone to drought.

Drought stress activates complex network regulatory mechanisms (Michaletti et al., 2018), which lead to changes in gene expression and biochemical and molecular processes in plants (Golldack et al., 2014). Under soil water deficiency, the optimal water supply is balanced in plant tissues, cell hydration is maintained, and water loss is avoided. The plant accumulates stress-protective metabolites (e.g., tryptophan, trehalose, flavonoids, glutathione, etc.) to prevent acute cell damage and membrane integrity by triggering the antioxidant system and deploying peroxidases (Cramer et al., 2007). Stress-related transcription factors (TFs), such as the MYB, bHLH, NAC, AP2/ERF, NFY and HSF, which specifically bind to *cis*-regulatory elements to activate downstream gene expression in response to stress signals, can be activated under drought stress (Hu et al., 2022). The mechanisms of plants in response to alternating or extreme drought and rehydration have become a research focus in several fields, including environmental and ecological preservation, genetics and breeding improvement (AbdElgawad et al., 2015; Zhao et al., 2022). The physiological and molecular functions in plants can be recovered after rehydration. However, the compensation of rehydration to plant growth after drought stress is often limited. Additionally, large-scale studies using high-throughput genomics have revealed the responses of metabolite accumulation or transcript changes in higher plants under drought and rehydration conditions (Chen et al., 2020; Sousa et al., 2022). However, there is a lack of comprehensive understanding of the mechanisms underlying drought tolerance in terms of transcriptomic and metabonomic integration, especially in endemic plants in karst regions.

*Illicium difengpi* (Schisandraceae) is a perennial evergreen shrub of *Illicium*, subg. *Cymbostemon*, distributed in the karst region of Guangxi Province with small and isolated populations. *I. difengpi* has high ornamental value, and the stem and root bark of this plant are used for traditional Chinese medicine, but the species is listed on the National Key Protection Plant List (Category II) (http://www.iplant.cn/rep/protlist). *I. difengpi* plants show strong drought resistance and can survive in rocky areas with an elevation of 200~1200 meters, such as on mountain tops, in rocky crevices with soil, or under sparse woodlands on rocky mountains. The leaves of this species have morphological characteristics that enable adaptation to drought stress, such as well-developed leaf veins, thick cuticles and well-developed palisade tissue (Kong et al., 2012). As a drought-tolerant plant, *I. difengpi* has demonstrated strong adaptability to various abiotic stresses, including drought and high temperatures. This makes it a valuable but understudied nonmodel plant resource that can enhance our understanding of the potential molecular mechanisms underlying adaptation and tolerance to drought stress. Research on the drought tolerance of *I. difengpi* has mainly focused on physiological mechanisms (Meng et al., 2019; Wang et al., 2021), few studies having investigated the molecular mechanisms of its adaptation to drought stress (Liu et al., 2022). The drought tolerance of *I. difengpi* has rarely been studied from an omics perspective. Therefore, the present study investigates the transcriptomic and metabolomic responses of the karst endemic medicinal plant *I. difengpi* to drought and rehydration treatment. The objective is to gain a full understanding of drought tolerance mechanisms in *I. difengpi* in terms of transcriptomic and metabolomic profiling.

## 2 Materials and methods

### 2.1 Plant materials and water treatments

**Figure 1:**
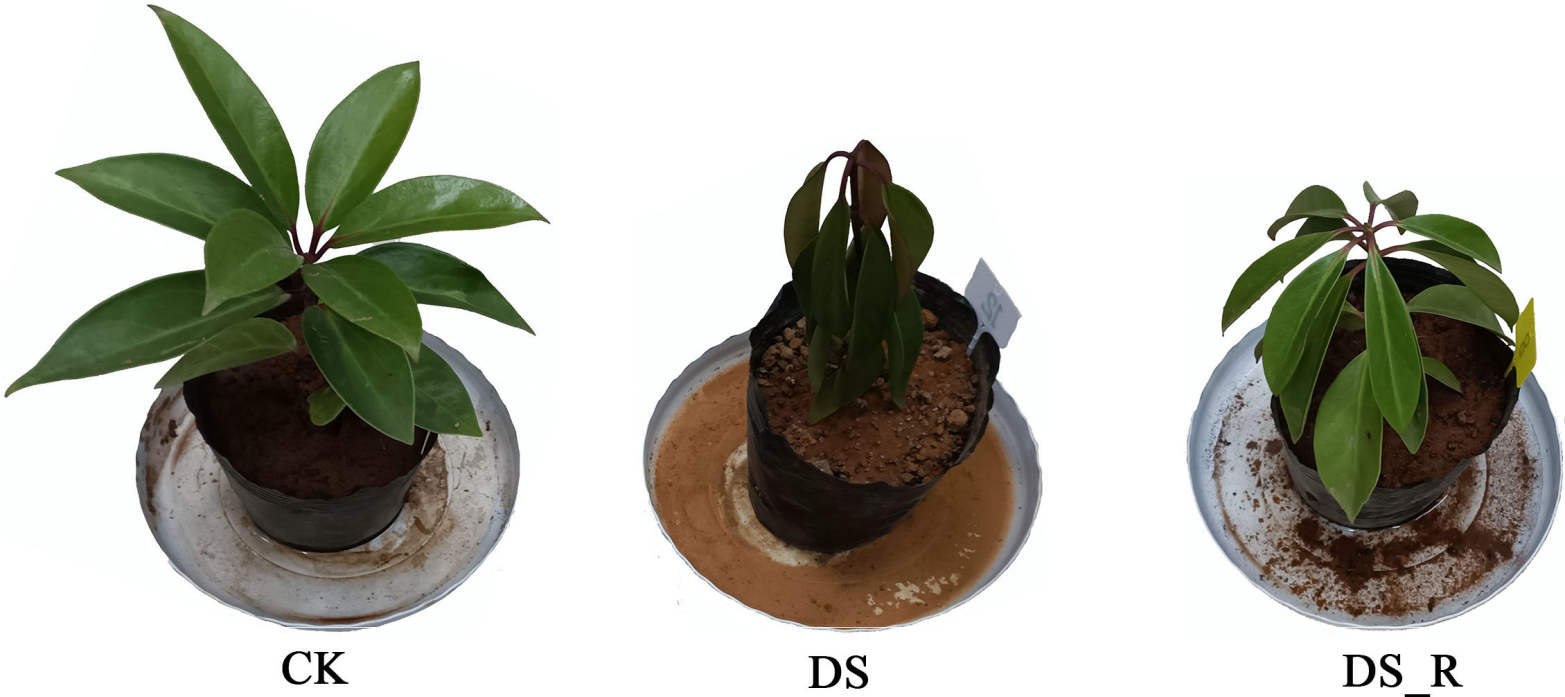
Morphological changes in *I. difengpi* plants during different treatments. CK, well-watered treatment; DS, drought stress treatment; DS_R, drought–rehydration treatment.

The experiment was performed at a karst germplasm nursery with a transparent rain shelter (25°4′N, 110°17′E) at the Guangxi Botanical Research Institute in Guangxi, China. Two-year-old plants from seeds collected in Jingxi County, Baise City, Guangxi Province, China, cultivated in plastic pots were subjected to water treatment. *I. difengpi* plants with consistent height (height: 22~24 cm) and leaf number (12~14 leaves) were selected. The plants were subjected to three soil water conditions for 18 days (**Figure 1**): well-watered treatment (50% of soil water content, water/dry soil, CK), drought stress treatment (10% of soil water content, water/dry soil, DS) and drought–rehydration treatment (rehydration to 50% of soil water content for 3 days after DS treatment for 15 days, DS_R), with four independent biological replicates for each group. After the drought and rehydration treatments, leaf samples (mature functional leaves, from the 2nd to 4th leaves from the top) were collected, frozen in liquid nitrogen, and stored at -80°C for transcriptome, quantitative real-time PCR (RT-qPCR) and metabolome analysis.

### 2.2 RNA isolation, generation of RNA-seq data and data processing

Total RNA was extracted from the *I. difengpi* samples with Trizol^®^ reagent (Invitrogen, Carlsbad, CA, USA) following the manufacturer’s instructions. The purity, quantity, and integrity of RNA were assessed on a NanoPhotometer spectrophotometer (IMPLEN, Los Angeles, CA, USA), Qubit RNA Assay Kit in a Qubit 2.0 Fluorometer (Life Technologies, Carlsbad, CA, USA), and RNA Nano 6000 Assay Kit for the Agilent Bioanalyzer 2100 system (Agilent Technologies, Santa Clara, CA, USA).

A total of 1 ug of RNA per sample was used as the input material for library preparation. The mRNA was purified from the total RNA using poly-T oligo-attached magnetic beads. Sequencing libraries from different treatments were constructed with the purified mRNA (100 ng) using the NEBNext^®^ UltraTM RNA Library Prep Kit for lllumina^®^ (NEB, USA) following the manufacturer’s instructions, and index codes were added to attribute sequences to each sample. Library sequencing was performed on an lllumina NovaSeq 6000 platform for generation of 150 bp paired end reads. The raw data of *I. difengpi* in this study have been deposited in the NCBI Sequence Read Archive (SRA) under bioproject accession number PRJNA983054.

Clean data were obtained by removing reads containing adapters with an N content greater than 10% and base quality (Q) less than or equal to 10 using the SeqPrep program (https://github.com/jstjohn/SeqPrep) and the Sickle program (https://github.com/najoshi/sickle). FastQC (v 0.12.1) (Andrews, 2010) was used to calculate the GC content, Q20 and Q30 of both the raw and clean data. The clean reads were then *de novo* assembled into unigene sequences using Trinity (v 2.6.6) (Grabherr et al., 2011), and a Python script was employed to extract the longest transcript for each gene as the reference sequence. The reference sequences were further compared against the Nr (Deng et al., 2006), Swiss-Prot (Apweiler et al., 2004), GO (Ashburner et al., 2000), COG (Tatusov et al., 2000), KEGG (Kanehisa et al., 2004) and Pfam (Finn et al., 2013) databases using BLAST (Altschul et al., 1990) for gene functional annotation. Finally, UpSetR (Conway et al., 2017) was utilized to visualize the annotations of these six databases for the genes.

The unigene sequences from each sample back to the reference sequences were mapped using Hisat2 (v 2.4) (Kim et al., 2013). The gene expression levels were calculated from the fragments per kilobase of transcript per million fragments mapped (FRKM) method (Mortazavi et al., 2008) using StringTie (v 1.3.1) (Pertea et al., 2015). DESeq2 (Anders and Huber, 2010) was used to determine the differentially expressed genes (DEGs) with a filtering threshold of fold change ≥2 and FDR < 0.01. The expression levels of the DEGs in each sample were standardized using the built-in *scale* function in R software (v 4.2.2). Principal component analysis (PCA) was performed using the built-in *prcomp* function, and cluster analysis was conducted using the *pheatmap* function to generate an expression heatmap. Gene otology (GO) term enrichment was carried out by mapping all genes to the corresponding terms in the GO database (http://www.geneontology.org/), and pathway analysis was performed based on the KEGG database (https://www.kegg.jp/) using hypergeometric testing to identify significantly enriched GO terms and pathways (*p* < 0.05) compared to the whole-genome background. Visualization of GO terms and pathways was performed using the R package ggplot2 (Villanueva and Chen, 2019) through Hiplot Pro (https://hiplot.com.cn/), a comprehensive web service for biomedical data analysis and visualization.

Reverse transcription was performed using the Monad First Strand cDNA Synthesis Kit. Six genes were selected to verify RNA-Seq data by RT-qPCR with *Actin* as a reference gene. Primers were designed using PRIMER-BLAST (Ye et al., 2012). RT-qPCR was carried out on an ABI7500 Real-Time PCR System (Applied Biosystems). The total reaction volume was 10 µL: 5 µL of MonScriptTM RTIII All-in-one Mix with dsDNase (Monad Biotech Co., Ltd.), 0.7 µL of each forward and reverse primer, 2.55 µL RNase-free water and 1 µL template DNA. The reaction conditions on the thermal cycler were as follows: 95°C for 2 min and 40 cycles of 95°C for 5 s and 60°C for 30 s. Four biological replicates were analyzed in independent runs. The quantification of gene expression levels was calculated as 2*^−^* ^ΔΔCt^ (Livak and Schmittgen, 2001) relative to the CK samples. The graphs for gene expression were prepared in Microsoft Excel 2007. All primers used in the present study are listed in **Supplementary Table S1**.

### 2.3 Sample extraction and metabolome profiling

The leaf samples of *I. difengpi* plants were freeze–dried using a SCIENTZ-100F freeze–dryer under vacuum. The dried samples were ground to a powder using an MM 400 grinder. One hundred milligrams of the powder was dissolved in 1 mL of 70% methanol extraction solution and kept overnight in a refrigerator at 4°C. The mixture was vortexed six times to ensure a thorough extraction during the process. The resulting extract was centrifuged at 12,000 rpm for 10 min, and the supernatant was collected after centrifugation. The supernatant was then filtered through a 0.22-μm-pore-size membrane filter and analyzed by UPLC-MS/MS (UPLC, SHIMADZU CBM30A, https://www.shimadzu.com/; MS/MS, QTRAP^®^ 4500+, https://sciex.com/).

The liquid phase conditions mainly included a Waters ACQUITY UPLC HSS T3 C18 column (1.8 µm, 2.1 mm×100 mm), with mobile phase A as ultrapure water (0.1% formic acid) and mobile phase B as acetonitrile (0.1% formic acid). The gradient elution was as follows: 0 min, water/acetonitrile (95:5 V/V); 10.0 min, water/acetonitrile (5:95 V/V); 11.0 min, water/acetonitrile (5:95 V/V); 11.1 min, water/acetonitrile (95:5 V/V); and 15.0 min, water/acetonitrile (95:5 V/V). The flow rate was 0.4 mL min^-1^, the column temperature was 40°C, and the injection volume was 2 μL.

The mass spectrometry conditions mainly included an electrospray ionization (ESI) source with a temperature of 550°C, positive ionization at 5500 V and negative ionization at -4500 V. The gas settings were as follows: gas I (GS I) at 55 psi, gas II (GS II) at 60 psi, curtain gas (CUR) at 25 psi, and collision-activated dissociation (CAD) parameters set to high. In the triple-quadrupole (QTRAP) system, each ion pair was scanned and detected based on the optimized declustering potential (DP) and collision energy (CE).

Using the NMDB and public databases (Ren et al., 2022), the *wiff* format raw data obtained from UPLC-MS/MS were subjected to qualitative and quantitative analysis using Analyst (v 1.6.3) (Ren and Chen, 2023). In the qualitative analysis, isotopic signals, duplicate signals of ions containing K^+^, Na^+^, and NH_4_^+^ ions, and duplicate signals of ions that were themselves fragments of larger molecules were removed using Microsoft Excel 2007. In the quantitative analysis, all chromatographic peaks in the samples were integrated using multiple reaction monitoring (MRM) mode based on triple-quadrupole mass spectrometry. The integration and calibration of the chromatographic peaks were performed using MultiaQuan (3.0.2, AB SCIEX, Concord, ON, Canada) to achieve metabolite quantification.

Differentially expressed metabolites (DEMs) were selected using the OPLS-DA function (OPLSR.Anal) in the MetaboAnalystR package in R software (v 4.2.2) (Chong and Xia, 2018), with a variable importance in projection (VIP) ≥ 1 and a fold-change of ≥ 1.5 (or ≤ 0.67). The identified DEMs were annotated using the KEGG compound database (https://www.kegg.jp/kegg/compound/) and were analyzed for pathway enrichment using the KEGG pathway database (https://www.kegg.jp/kegg/pathway.html). PCA was performed using the built-in *prcomp* function in R software (4.2.2), and the relative abundances of differentially abundant metabolites in each group were standardized using the built-in *scale* function. K-means clustering analysis was then performed using the R package Mfuzz (Kumar and Matthias, 2007) through Hiplot Pro (https://hiplot.com.cn/).

### 2.4 Conjoint analysis

The transcriptome and metabolome data were standardized (unit variance scaling) and subjected to statistical analysis to determine their relationships under different treatments. Combining functional analysis, correlation analysis, metabolic regulatory pathways, and functional annotation analysis, key genes or metabolic regulatory pathways involved in the drought resistance mechanism were identified. Genes associated with secondary metabolite biosynthesis and metabolic pathways were taken for analysis. The standardized data were analyzed for correlations using the *cor* function in R software (4.2.2) with a Pearson’s correlation coefficient (|PCC|) threshold of ≥ 0.8 and *p* value ≤ 0.05. Data were organized using Microsoft Excel 2007, and gene expression and metabolite abundance relationships were visualized using Microsoft PowerPoint 2007 and TBtools (v 1.120) (Chen et al., 2018).

## 3 Results

### 3.1 *De novo* assembly and annotation

**Figure 2:**
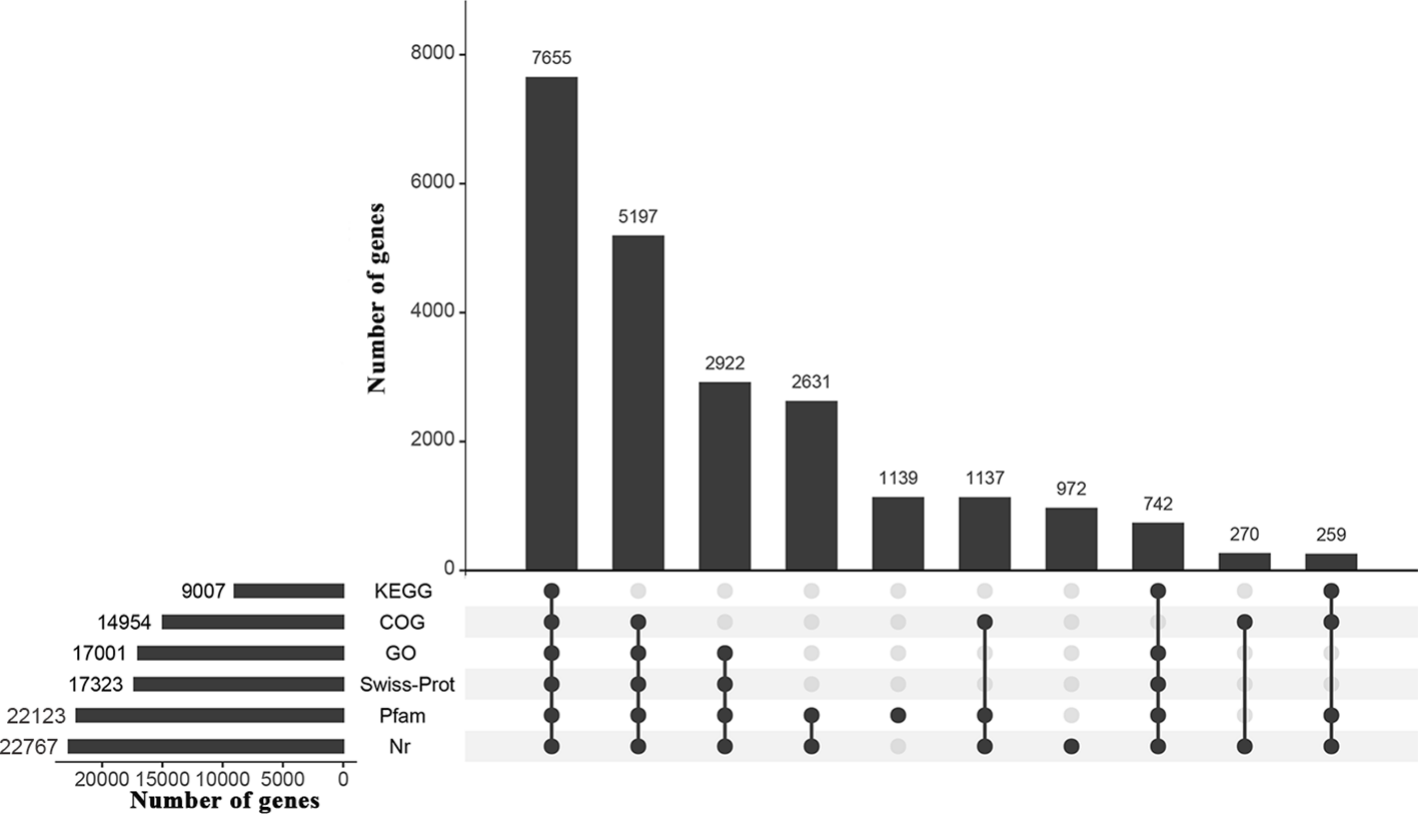
UpSet graph showing annotation results of the *I. difengpi* transcriptome against six databases. Nr, non-redundant protein sequence; Swiss-Prot, a manually annotated, non-redundant protein sequence; GO, gene ontology; COG, clusters of orthologous groups of proteins; KEGG, kyoto encyclopedia of genes and genomes; Pfam, homologous protein family.

All transcriptome analyses of the three water treatments, CK, DS and DS_R, resulted in a total of 78.20 Gb clean data from 12 samples, with Q20 > 97%, Q30 > 93% and GC content > 46% (**Supplementary T** **able S2**). *De novo* assembly generated 231,784 unigene sequences with an average length of 689.19 bp and an N50 of 1,255 bp. The average length of the reference sequence was 552.63 bp, with an N50 length of 712 bp. About 71.09% of the total reads were mapped back onto the reference sequence (**Supplementary Tables S2, S3**), indicating good sequencing quality. A total of 24,488 genes in the reference sequence were annotated through BLAST analysis against the Nr (22,767), Swiss-Prot (17,323), GO (17,001), COG (14,954), KEGG (9,007) and Pfam (22,123) databases (**Figure 2**).

A total of 8,739 DEGs in response to CK, DS and DS_R treatment were generated using a stringent threshold fold change ≥ 2 and FDR < 0.01. Both the DS and DS_R treatments induced more upregulated DEGs than downregulated DEGs. Specifically, DS treatment induced 3,301 upregulated DEGs and 2,273 downregulated DEGs, while DS_R treatment induced 2,319 upregulated DEGs and 1,587 downregulated DEGs (**Supplementary F** **igure S1**).

**Figure 3:**
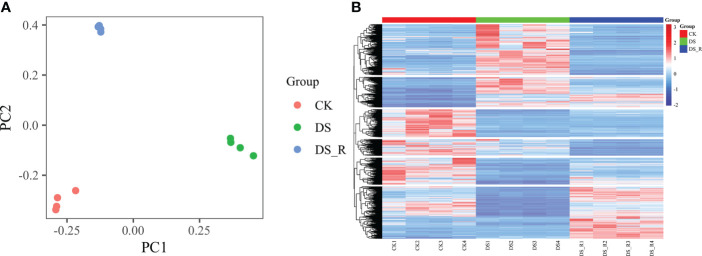
Global gene expression profiling of leaves under three soil water treatments. **(A)** Principal component analysis (PCA) of RNA-seq data obtained from leaves of *I difengpi* well-watered (CK), drought stress (DS) and drought–rehydration (DS_R) treatments. **(B)** Hierarchical clustering heatmap of RNA-seq data. The abscissa represents the sample names and hierarchical clustering results, whereas the ordinate represents DEGs and hierarchical clustering results. The expression intensity is indicated as the scale bar, red shading indicates high expression, and blue shading indicates low expression.

The PCA results (**Figure 3A**) showed distinct gene expression patterns among the different treatment groups. According to PC1, the gene expression under the DS_R treatment was closer to that under the CK treatment, accounting for 47.59% of the total data variation, while PC2 accounted for 30.65% of the total data variation. The hierarchical cluster heatmap of DEGs (**Figure 3B**) demonstrated significant changes in gene expression patterns during drought stress, with 65.25% of the genes showing a tendency of stable expression after rehydration but also the emergence of some new differentially expressed genes.

To validate the RNA-seq data, RT-qPCR was carried out with 6 genes of interest. (**Supplementary T** **able S4**). These genes greatly differed in expression between the three treatments, ranging from 1 to 600 reads per kilobase transcript per million reads (FRKM). The expression levels of the genes determined by RT-qPCR correlated well with those obtained from RNA-seq (R^2 = ^0.87) (**Supplementary Figure S2**), confirming the reliability of the DEGs identified by analyzing the RNA-seq data.

### 3.2 GO function analysis and KEGG analysis of DEGs

**Figure 4:**
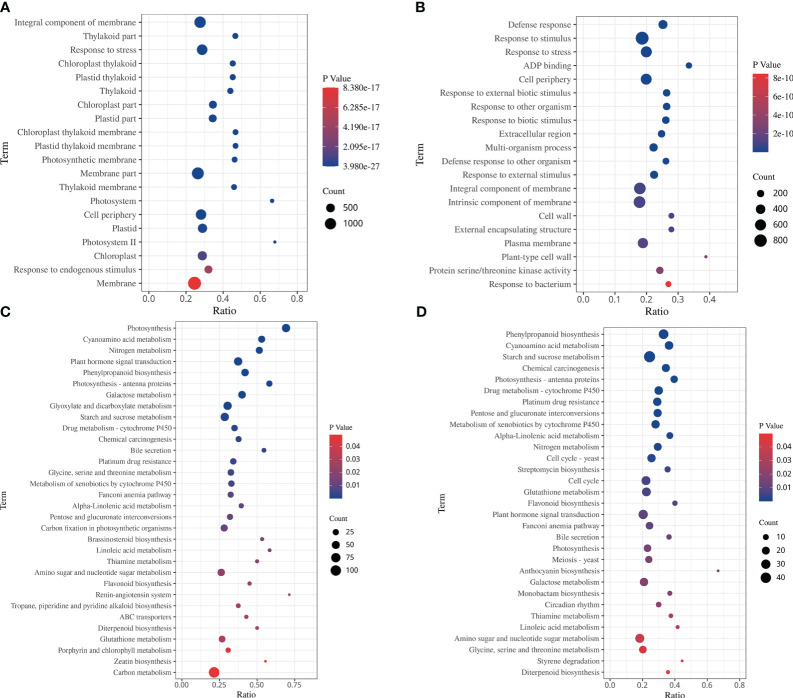
Top 20 GO terms and KEGG enrichment of DEGs under soil water treatments. **(A, C)** drought stress treatment. **(B, D)** drought–rehydration treatment. The abscissa represents the ratio of the number of different genes enriched in a pathway to the total number of annotated genes, where a greater ratio corresponds to greater enrichment. Deeper blue shading indicates greater statistical significance in terms of enrichment.

The GO enrichment analysis showed that the DEGs in response to drought stress and rehydration were categorized into 52 functional terms in three categories. Among them, genes associated with metabolic process and cellular process in the category “biological process”; cell, cell parts and organelle in the category “cellular components”; and binding and catalytic activity in the category “molecular function”, were the most abundant (**Supplementary Figure S3**). Among the 20 GO terms with the lowest *p* values in the enrichment analysis, the functional categories in the DS and DS_R treatments were mostly related to chloroplast and photosynthetic components (**Figures 4A, B**). In the DS treatment, the top 5 enriched biological processes were integral component of the membrane, thylakoid part, stress response, chloroplast thylakoid and plastid thylakoid, while under the DS_R treatment, the main enriched categories were defense response, response to stimulus, stress response, ADP binding and cell periphery.

The KEGG enrichment analysis showed 32 and 31 significantly enriched pathways under the DS and DS_R treatments, respectively (**Figures 4C, D**, *p* < 0.05). Among them, carbon metabolism, phytohormone signal transduction, glyoxylate and dicarboxylate metabolism, starch and sucrose metabolism and photosynthesis were the pathways with the most DEGs under the DS treatment. In the DS_R treatment, starch and sucrose metabolism, phenylpropanoid biosynthesis, amino sugar and nucleotide sugar metabolism, and the cell cycle were the main pathways. In addition, some enriched pathways were shared in common by the DS and DS_R treatments, including glutathione metabolism, flavonoid biosynthesis, nitrogen metabolism, photosynthesis, phenylpropanoid biosynthesis, cyanoamino acid metabolism, photosynthesis-antenna proteins and galactose metabolism. These results indicated that *I. difengpi* responds to drought stress and rehydration by enhancing its energy metabolism, reducing photosynthesis, regulating metabolic pathways and synthesizing metabolites.

### 3.3 Response TFs under drought and rehydration treatments

**Figure 5:**
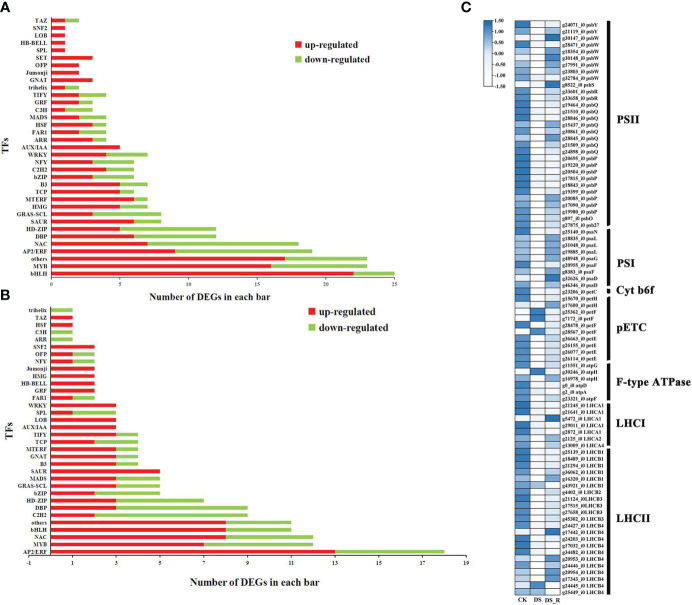
Transcription factors (TFs) responsive to drought stress **(A)** and drought–rehydration **(B)** treatment. **(C)** DEGs involved in photosynthesis and photosynthesis-antenna proteins.

A total of 249, 162 and 231 DEGs were identified as encoding TFs in the CK_vs_DS (162 upregulated and 87 downregulated), CK_vs_DS_R (104 upregulated and 58 downregulated) and DS_vs_DS_R comparisons (114 upregulated and 117 downregulated), which could be assigned to 27, 15 and 25 families, respectively (**Supplementary Table S5**). Compared to the CK treatment, the DS treatment induced the upregulation of several members of the ARR, HSF, bHLH, GRF, B3, MYB, TCP, C2H2 and WRKY TF families (**Figures 5A, B**). Most members of the MYB, WRKY, bHLH, GRF and B3 gene families continued to be upregulated under DS_R treatment. Additionally, most members of the NAC and AP2/ERF TF families were also upregulated by DS_R treatment.

### 3.4 DEGs involved in photosynthesis under drought and rehydration treatments

A total of 86 DEGs were annotated to the photosynthesis and photosynthesis-antenna protein pathways in the DS and DS_R treatments (**Figure 5C**). Among these, genes related to psaN, psaL, psaG, psaF, psaD, psbY, psbW, psbS, psbR, psbQ, psbP, psbO and psb27 in both photosystem I (PSI) and photosystem II (PSII) were downregulated, and genes related to the photosynthetic electron transport chain (pETC), such as petH, petF and petE, were downregulated under the DS treatment. Additionally, genes related to the cytochrome b6/f complex (Cyt b6f), the F-type ATPase, and the light-harvesting chlorophyll II protein complex, were mostly significantly downregulated under the DS treatment. However, DEGs related to photosynthesis and photosynthesis-antenna protein were mostly significantly upregulated after the DS_R treatment.

### 3.5 Metabolomics analysis

Metabolomic analysis revealed a total of 632 metabolites in the leaves of *I. difengpi* plants under the CK, DS and DS_R treatments, including 351 in the positive model and 281 in the negative model. The complete list of detected metabolites, along with their corresponding relative peak areas, can be found in **Supplementary T** **able S6**. Based on the hierarchical classification of metabolites, the majority of detected compounds were flavones, phenolic acids, flavonols, amino acids and derivatives, saccharides and alcohols (**Supplementary Figure S4**).

**Figure 6:**
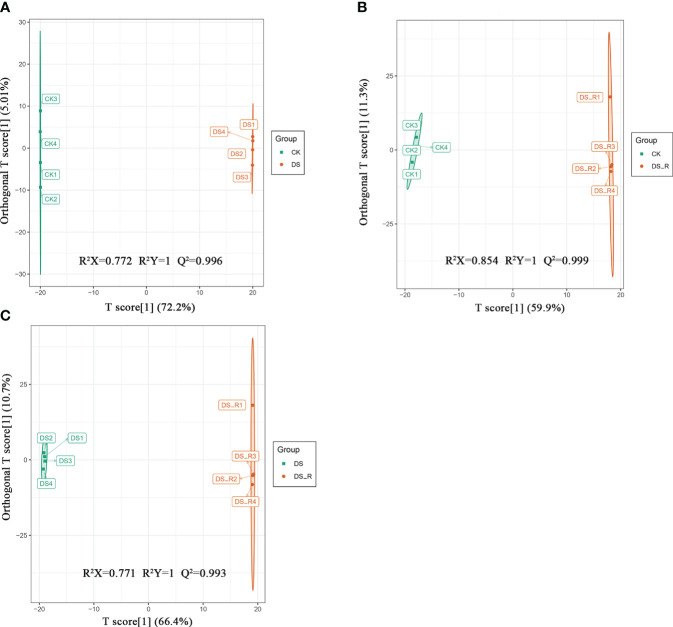
OPLS-DA model plots of the metabolites identified between pairs of groups of *I difengpi*. **(A)** Well-watered (CK)_vs_drought stress (DS). **(B)** Well-watered (CK)_vs_Drought–rehydration (DS_R). **(C)** Drought stress (DS)_vs_Drought–rehydration (DS_R). R^2^: The interpretation rate of the model to the matrix; Q^2^: The prediction ability of the model.

A total of 427 differentially expressed metabolites (DEMs) were identified using the criteria VIP ≥ 1 and fold change ≥ 1.5 or ≤ 0.67. As shown in **Supplementary Figure S5**, there were 223 and 108 upregulated DEMs and 115 and 115 downregulated DEMs in the DS and DS_R treatments, respectively. In both the DS and DS_R treatments, flavonols and phenolic acids were the main accumulated metabolites in *I. difengpi*. The percentages of flavonols (15.71%) and phenolic acids (9.67%) in the DS treatment were higher than those in the DS_R treatment (10.00% and 9.13%) (**Supplementary Figures S6A, B**). The OPLS-DA model showed that the prediction parameters R^2^X, R^2^Y and Q^2^ were all higher than 0.7, 1 and 0.9, respectively, indicating the reliability and stability of the OPLS-DA model (**Figure 6**).

**Figure 7:**
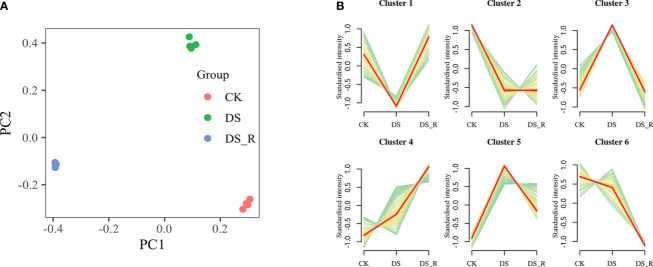
Metabolism analysis of leaves under the three soil water treatments. **(A)** PCA of metabolite data obtained from leaves of *I difengpi* plants under well-watered (CK), drought stress (DS) and drought–rehydration treatments (DS_R). **(B)** Differential metabolite K-means diagram. The x-coordinate represents the sample, and the y-coordinate represents the relative content of the standardized metabolite.

PCA showed clear differences between the CK, DS and DS_R treatments, and the samples of the DS and DS_R treatments were more similar, indicating a high degree of similarity in the metabolomic features between these two treatments (**Figure 7A**). PC1 and PC2 accounted for 92.10% of the total variation, with PC1 (58.10%) explaining the differences between the DS and DS_R treatments and PC2 (34.00%) explaining the general direction of changes in metabolite concentrations under the DS and DS_R treatments.

K-means clustering analysis grouped the 427 DEMs into 6 clusters (clusters 1-6, **Figure 7B**). Cluster 1 showed a decrease in the content of 47 metabolites under the DS treatment, which was restored to CK levels under the DS_R treatment. Cluster 3 exhibited an accumulation of 125 metabolites under the DS treatment, including l-isoleucine, l-leucine, l-serine, quercetin, spermidine and dihydroquercetin, which were restored to the CK level under the DS_R treatment. Clusters 4 and 5 demonstrated an accumulation of 146 metabolites under both DS and DS_R treatments, such as putrescine, l-asparagine, l-tryptophan, glutathione, l-ascorbate, d-sucrose, d-sorbitol and d-trehalose. Clusters 2 and 6 showed a decrease in the contents of 109 metabolites, including naringenin, raffinose and d-inositol, under both DS and DS_R treatments (**Supplementary Table S7**).

**Figure 8:**
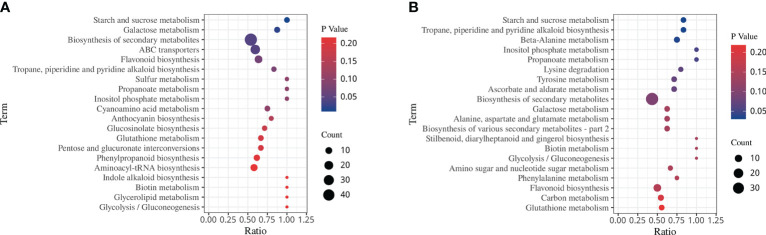
KEGG enrichment of DEMs under the three soil water treatments. **(A)** Drought stress treatment. **(B)** Drought–rehydration treatment. The abscissa represents the ratio of the number of different metabolites enriched in a pathway to the total number of metabolites, where a greater ratio corresponds to greater enrichment. Deeper blue shading indicates greater statistical significance in terms of enrichment.

KEGG analysis showed 4 and 3 significantly enriched pathways involved in drought stress and rehydration, respectively (**Figures 8A, B**, *p* < 0.05). Specifically, there was significant enrichment in starch and sucrose metabolism, galactose metabolism, biosynthesis of secondary metabolites and ABC transporters under the DS treatment. However, under the DS_R treatment, there was significant enrichment of tropane, piperidine, and pyridine alkaloid biosynthesis, beta-alanine metabolism and starch and sucrose metabolism. In particular, four significantly enriched pathways (inositol phosphate metabolism, galactose metabolism, flavonoid biosynthesis and glutathione metabolism) were all found under both DS and DS_R treatments, indicating their close association with the response of *I. difengpi* to drought stress and rehydration.

### 3.6 Correlation analysis of transcriptome and metabolome data

To screen metabolites and genes in response to drought stress and rehydration, correlation analysis was performed between DEGs and DEMs (|PCC| ≥ 0.8, *p* ≤ 0.05). A total of 1,106 DEGs were found to be correlated with 331 DEMs during drought stress, while 776 DEGs were correlated with 230 DEMs under rehydration. Additionally, common pathways were obtained through the KEGG database for DEGs and DEMs. **Supplementary Figure S7** shows that, compared with under the CK treatment, DEGs and DEMs under the DS and DS_R treatments were mapped to 71 and 64 pathways, respectively. Among them, the starch and sucrose metabolism pathways (*p* < 0.05) were significantly enriched under the DS and DS_R treatments. Galactose metabolism, flavonoid biosynthesis, glutathione metabolism and amino acid biosynthesis were also enriched under the DS and DS_R treatments, indicating that these pathways play important roles in *I. difengpi* in response to drought stress and rehydration.

**Figure 9:**
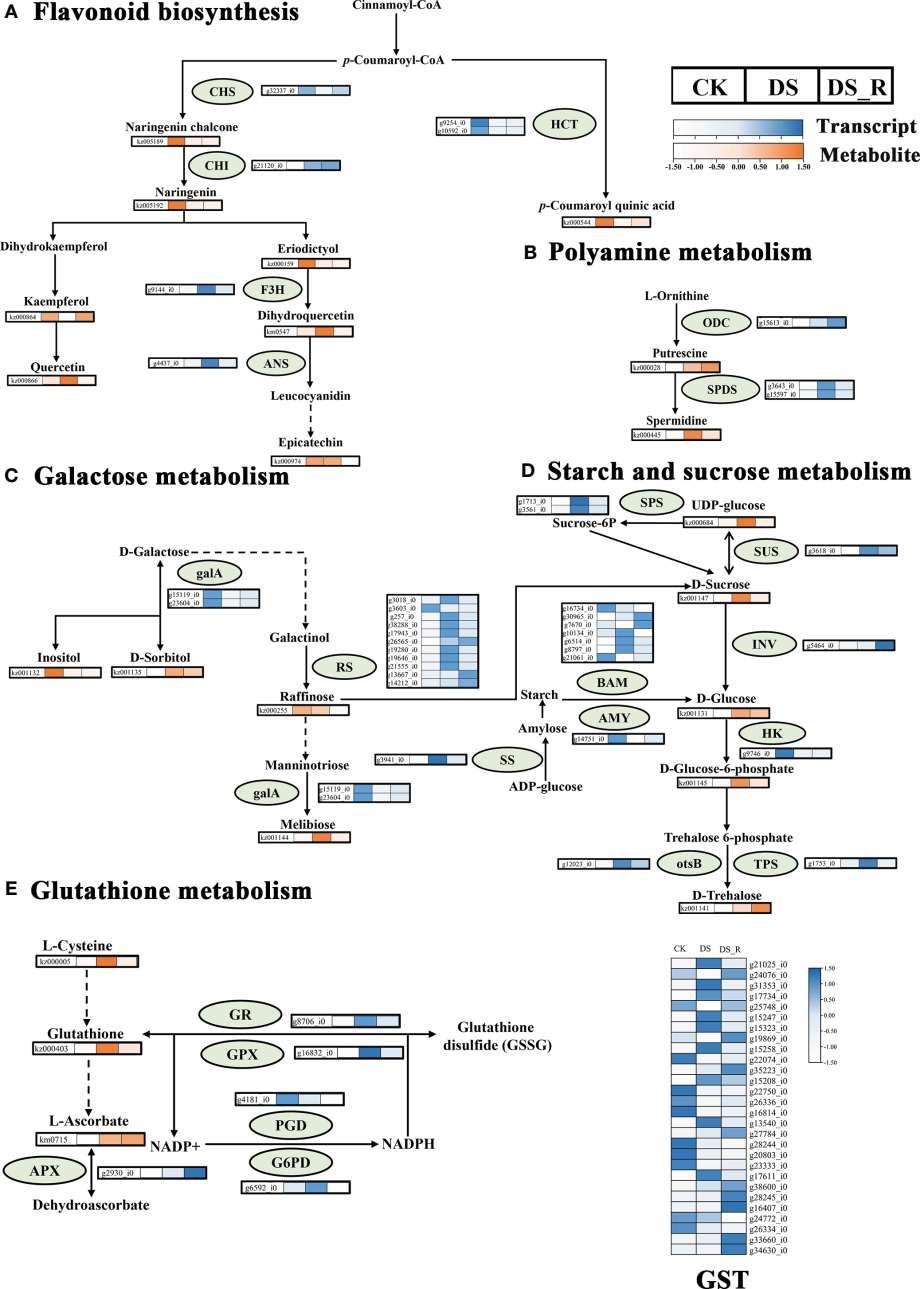
Changes in key enzyme activities at the transcript and metabolite levels in response to water treatments. **(A)** Flavonoid biosynthesis. **(B)** Polyamine metabolism; **(C)** Galactose metabolism. **(D)** Starch and sucrose metabolism. **(E)** Glutathione metabolism. HCT, shikimate *O*-hydroxycinnamoyl transferase; CHS, chalcone synthase; F3H, naringenin 3-dioxygenase; CHI, chalcone isomerase; ANS, anthocyanin synthase; SPDS, spermidine synthase; ODC, ornithine decarboxylase; INV, beta-fructofuranosidase; otsB, trehalose 6-phosphate phosphatase; TPS, trehalose phosphate synthase; SUS, sucrose synthase; SPS, sucrose-phosphate synthase; SS, starch synthase; galA, alpha-galactosidase; HK, hexokinase; RS, sucrose galactosyltransferase; BAM, beta-amylase; AMY, alpha-amylase; GST, glutathione S-transferase; GR, glutathione reductase; GPX, glutathione peroxidase; PGD, 6-phosphogluconate dehydrogenase; G6PD, glucose-6-phosphate 1-dehydrogenase; APX, l-ascorbate peroxidase. Dotted arrows indicate multiple enzyme-catalyzed reaction steps. Colored boxes represent the normalized intensity of transcript levels (in blue) and metabolite levels (in orange) across different water treatments. CK, well-watered treatment; DS, drought stress treatment; DS_R, drought–rehydration treatment.

The correlation analysis between DEGs and DEMs in the flavonoid synthesis pathway (**Figure 9A**) revealed that 6 genes were related to flavonoid synthesis. Among these, the *g9144_i0* gene (naringenin 3-dioxygenase, F3H, EC: 1.14.11.9) was upregulated under DS treatment, but downregulated after DS_R treatment, which promoted dihydroquercetin accumulated under DS treatment but recovered under DS_R treatment. Two genes, including the *g10592_i* gene (shikimate *O*-hydroxycinnamoyl transferase, HCT, EC: 2.3.1.133) were downregulated under both DS and DS_R treatments, which decreased coumaroyl quinic acid content. The *g21120_i* gene (chalcone isomerase, CHI, EC: 5.5.1.6) was upregulated under both DS and DS_R treatments, which decreased naringenin content. The *g32337_i0* gene (chalcone synthase, CHS, EC: 2.3.1.74) was downregulated under DS treatment but upregulated after DS_R treatment, which decreased naringenin chalcone content. Additionally, the *g4437_i0* gene (anthocyanidin synthase, ANS, EC: 1.14.11.9) was upregulated under DS treatment but downregulated after DS_R treatment.

The integrated analysis of the drought stress and rehydration response of *I. difengpi* also showed changes in polyamine metabolism, specifically regarding putrescine and spermidine. As shown in **Figure 9B**, a total of 3 genes were related to polyamine biosynthesis. The *g15613_i0* gene (ornithine decarboxylase, ODC, EC: 4.1.1.17) was upregulated under both DS and DS_R treatments. This increase in ODC levels could lead to the observed putrescine accumulation. Also, two genes coding for spermidine synthase (SPDS, EC: 2.5.1.16), which catalyzes the conversion of putrescine into spermidine, were upregulated under DS treatment but downregulated during DS_R treatment, correlating with similar changes in spermidine levels.

As shown in **Figure 9C**, a total of 13 genes were related to galactose metabolism. Two genes, including the *g15119_i0* gene (alpha-galactosidase, galA, EC: 3.2.1.22) were downregulated under both DS and DS_R treatments, which promoted d-sorbitol and melibiose accumulated under DS treatment, but decreased inositol content. Eleven genes, including the *g19280_i0* gene (raffinose synthase, RS, EC: 2.4.1.82), were mostly upregulated under DS treatment but downregulated after DS_R treatment, which decreased raffinose content.

As shown in **Figure 9D**, a total of 16 genes were related to starch and sucrose metabolism. Among these, the *g5464_i0* gene (beta-fructofuranosidas, INV, EC: 3.2.1.26) was upregulated under DS_R treatments, which promoted d-glucose accumulated. The *g9746_i0* gene (hexokinase, HK, EC: 2.7.1.1) was downregulated under both DS and DS_R treatments, which promoted the accumulated of d-glucose-6-phosphate. The *g12023_i0* gene (trehalose 6-phosphate phosphatases, otsB, EC: 3.1.3.12) and the *g1753_i0* gene (trehalose phosphate synthase, TPS, EC: 2.4.1.15 3.1.3.12), were upregulated under DS treatment, but the g*1753_i0* gene showed stable expression after DS_R treatment, promoted the accumulated of d-trehalose. The *g3618_i0* gene (sucrose synthase, SUS, EC: 2.4.1.13) was upregulated under both DS and DS_R treatments, which promoted UDP-glucose accumulated. Additionally, 7 genes (beta-amylase, BAM, EC: 3.2.1.2) related to starch degradation, were mostly upregulated under DS treatment, while genes related to starch synthesis, such as sucrose-phosphate synthase (SPS, EC: 2.4.1.14) and starch synthase (SS, EC: 2.4.1.21) were upregulated under DS treatment but downregulated during DS_R treatment. The *g14751_i0* gene (alpha-amylase AMY, EC: 3.2.1.1) was downregulated under both DS and DS_R treatments.

The metabolomics analysis showed that glutathione, l-cysteine and l-ascorbate were involved in glutathione metabolism. Five genes were related to glutathione metabolism (**Figure 9E**). The *g2930_i0* gene (l-ascorbate peroxidase, APX, EC: 1.11.1.11) was upregulated under both DS and DS_R treatments, which promoted the accumulated of l-ascorbate. The *g8706_i0* gene (glutathione reductase, GR, EC: 1.8.1.7) and the *g16832_i0* gene (glutathione peroxidase GPX, EC: 1.11.1.9) were upregulated under DS treatment but downregulated after DS_R treatment, which promoted glutathione accumulated under DS treatment but recovered during DS_R treatment. The *g4181_i0* gene (6-phosphogluconate dehydrogenase, PGD, EC: 1.1.1.44) was downregulated under both DS and DS_R treatment. The *6592_i0* gene (glucose-6-phosphate 1-dehydrogenase, G6PD, EC: 1.1.1.49) was upregulated under DS treatment but recovered after DS_R treatment. Additionally, 28 genes (S-transferase, GST, EC: 2.5.1.18), most of which were upregulated under DS treatment but recovered during DS_R treatment.

Four amino acid metabolism pathways were regulated under drought stress and rehydration (**Supplementary Table S8**). In the alanine, aspartate and glutamate metabolism, the *g8114_i0* gene (asparagine synthesis, asnB, EC: 6.3.5.4) was upregulated under both DS and DS_R treatments, which promoted l-asparagine accumulated. In the valine, leucine and isoleucine biosynthesis, the *g11815_i0* gene (branched-chain amino acid amino transfer, ilvE, EC: 2.6.1.42) was downregulated under both DS and DS_R treatments, which promoted l-leucine and l-isoleucine accumulated. In the phenylalanine, tyrosine and tryptophan biosynthesis, the *g13599_i0* gene (tryptophan synthase alpha chain, trpA, EC: 4.2.1.20) and the *g9790_i0* gene (tryptophan synthase beta chain, trpB, EC: 4.2.1.20) were downregulated under both DS and DS_R treatments, which promoted l-tryptophan accumulated. In the glycine, serine and threonine metabolism, the *g2861_i0* gene (serine-glyoxylate transaminase, AGXT, EC: 2.6.1.45) was downregulated under DS treatment but upregulated after DS_R treatment, which promoted l-serine accumulated under DS treatment and recovered during DS_R treatment.

## 4 Discussion

*I. difengpi* is a naturally drought-tolerant nonmodel plant and an important untapped genetic resource for studying the genetic and metabolic mechanisms underlying drought adaptation. In the present study, transcriptomic and metabolome analytical methods were combined to identify differentially expressed genes and metabolites under drought and rehydration conditions, and we aimed to preliminarily elucidate the molecular mechanisms of the response of *I. difengpi* to drought stress. Next-generation sequencing (NGS) technology, known for its accuracy, speed and low cost, is commonly used to explore unique genetic characteristics in both model and nonmodel plant systems (Unamba et al., 2015; Ren et al., 2022). The GC content represents the stability of DNA as well as the composition of genes and genomes, reflecting evolution, gene structure and gene regulation (Carels et al., 1998), and changes in GC content may reveal adaptability to different climatic conditions. The results in **Supplementary Table S2** show that the GC content of the *I. difengpi* transcriptome was 46.59%. This result is similar to previously published data (46.00~48.48%) (Liu et al., 2022). Similarly, in other studies on dicotyledonous plants, the GC content of *Picrorhiza kurrooa* was 44.6% (Gahlan et al., 2012) and that of *Nicotiana benthamiana* was 41.0% (Nakasugi et al., 2013).

Drought can induce the response of various genes, with more upregulated genes than downregulated genes (Wang and Gong, 2017). In the present study, the number of upregulated genes in *I. difengpi* under DS and DS_R treatments was greater than the number of downregulated genes (**Supplementary Figure S1**), indicating that *I. difengpi* primarily responds to drought stress and rehydration through the upregulation of genes. Upregulation of genes is likely a strategy employed by *I. difengpi* to actively adapt to environmental stress, while downregulation of gene expression helps reduce metabolism, inhibit growth and adapt to adverse conditions. The rehydration process compensates for the damage caused by drought, and the compensatory effect is also influenced by various factors, such as the degree of damage, rehydration time and growth condition of the damaged plant itself. Furthermore, the rehydration treatment reduced the number of DEGs in *I. difengpi* by 29.92% (**Supplementary Figure S1**), indicating a certain compensatory effect of rehydration, but not complete restoration, and resulting in some new DEGs. The hierarchical clustering heatmap of DEGs shown in **Figure 3B** illustrates that under rehydration treatment, 65.25% of the genes tended to have stable expression. Taken together, these results indicate that *I. difengpi* actively adapts to or resists drought through upregulated gene expression and exhibits a certain compensatory effect after rehydration, which may partly explain the extreme drought tolerance of *I. difengpi*.

TFs are important upstream regulatory proteins that play a crucial role in plant responses to abiotic and biotic stress (Ning et al., 2011). **Figure 5A** shows that most members of the six TF families (bHLH, HSF, MYB, ARR, WRKY and C2H2) were upregulated under drought stress treatment, indicating their potential role in positive regulation. The positive regulatory roles of HSF, WRKY, MYB, bHLH, ARR and C2H2 in the plant response to drought stress have been demonstrated in previous studies (Kang et al., 2013b; Yang and Lv, 2022). Additionally, most members of the GRF, TCP and B3 TF families were upregulated, and these TFs are known to regulate various growth and developmental processes, such as leaf development, flower symmetry and bud branching (Liu et al., 2014; Parapunova et al., 2014; Zhang et al., 2014). Furthermore, after rehydration treatment, most members of the MYB, WRKY, bHLH, GRF and B3 gene families continued to be upregulated, suggesting their involvement in regulating the recovery growth of *I. difengpi*.

Under drought stress, stomatal closure interrupts the supply of carbon dioxide to mesophyll cells, inhibiting carbon assimilation and light reactions, leading thereby to a decrease in photosynthetic efficiency (Chaves et al., 2009). The results of this study showed that under drought treatment, the majority of genes involved in PSI, PSII, Cyt b6f, pETC, F-type ATPase and light-harvesting chlorophyll II protein were downregulated (**Figure 5C**). Furthermore, GO enrichment analysis indicated that the top 20 enriched GO terms were mostly related to photosynthesis (**Figures 4A, B**), suggesting the inhibition of photosynthesis in *I. difengpi* leaves under drought stress. This further supports the idea that *I. difengpi*, experiencing drought stress, induces stomatal closure to reduce transpiration and consequently decreases photosynthetic capacity. Similar findings have been reported in wheat (Lv et al., 2022). When *I. difengpi* plants were watered again, a majority fraction of photosynthesis-related genes were upregulated, indicating gradual recovery of photosynthesis-related processes from drought stress after rehydration.

Despite the reduction in carbon fixation in leaves under DS treatment, plants accumulate large amounts of water-soluble carbohydrates such as glucose, fructose, sucrose and sorbitol to maintain cell turgor and protect their membrane integrity (Yang et al., 2019). Sucrose is the main soluble product of plant photosynthesis and it is an important regulatory factor in plant cellular metabolism (Xie et al., 2002). The results of the present study showed that under drought stress, genes related to d-sucrose synthesis, such as *SPS*, were upregulated, while genes related to d-sucrose hydrolysis, such as *INV*, were downregulated, promoted the accumulation of d-glucose (**Figure 9D**). Similar results have been reported in apple trees (Yang et al., 2019). In addition, DS treatment upregulated the expression of *TPS* gene and promoted the accumulation of d-trehalose in *I. difengpi*. TPS converts UDP-glucose into trehalose, thus enhancing the ability of *Selaginella pulvinata* to cope with adverse environments (Seki et al., 2007). Furthermore, *I. difengpi* exhibited higher levels of d-sorbitol under drought conditions. Research by Ranney et al. (1991) found that the osmoregulatory effect of cherry (*Prunus cerasus* and *P. avium* × *pseudocerasus*) under drought stress is mainly driven by sorbitol.

Starch is the main storage carbohydrate in plants (Ballicora et al., 2004). Under drought stress, starch gets hydrolyzed to glucose molecules (such as glucose-6-P and glucose-1-P). These molecules acts as a source of energy through respiration and precursors for the synthesis of sucrose (Geigenberger et al., 1997; Thalmann et al., 2016). The results of this study showed that drought stress downregulated the expression of *AMY* gene and upregulation the expression of *BAM* gene (**Figure 9D**). The concerted action of AMY and BAM promotes starch degradation, providing energy for plants to withstand drought stress. In summary, under drought treatment, photosynthesis in *I. difengpi* gradually slows, leading to a decrease in the quantity of photosynthetic products. This, in turn, increases the synthesis of d-sucrose, d-glucose, d-sorbitol and d-trehalose, regulating cell osmotic potential and water balance, while stored starch is degraded to provide energy, thereby maintaining normal physiological activities.

Under drought stress, plants produce a large amount of reactive oxygen species (ROS). These excessive ROS can cause damage to plant cells and, in severe cases, lead to plant death (Baxter et al., 2014). To sustain their growth and development, plants respond to drought stress by inducing the expression of genes involved in amino acid and secondary metabolite metabolism, producing large amounts of antioxidant substances such as glutathione, ascorbate, polyamines and flavonoids (Mittler et al., 2004; Alcázar et al., 2011; Nakabayashi et al., 2014).

Flavonoids possess strong antioxidant activity and can minimize the harmful effects of ROS on plants under drought stress (Ma et al., 2014). The major enzymes involved in the formation of various flavonoid compounds include CHS, CHI, F3’H, FLS, F3H, DFR, ANS and UGT. Among them, CHS, CHI, F3’H, FLS and F3H are responsible for the production of flavanols and other flavonoid compounds, while DFR, ANS and UGT are involved in anthocyanin accumulation in later stages (Khusnutdinov et al., 2021). The response of flavonoid content to drought stress varies between different species. Studies conducted in *Arabidopsis thaliana*, for example, have shown significant accumulation of dihydroflavonols and anthocyanins under drought stress (Nakabayashi et al., 2014; Zandalinas et al., 2016). Yang and Lv (2022) found that vitexin and naringenin were significantly increased in *Haloxylon amsenjimodendron* under drought stress. In our study, dihydroquercetin and quercetin significantly accumulated (**Figure 9A**) under drought stress treatment. Furthermore, genes related to CHI, F3H and ANS showed an upregulated expression under drought stresss treatment, with weaker levels or even downregulated during rehydration. The genes and metabolites related to flavonoids actively respond to drought stress by providing resistance to drought.

The ascorbate–glutathione cycle plays a crucial role in defending against oxidative damage caused by drought stress (Foyer and Noctor, 2011). The protective effect of the ascorbate–glutathione cycle against drought-induced oxidative damage is closely related to the gene expression of key enzymes involved in the regulation of the oxidative state, such as APX, GRC1, DHAR, MDHAR, GPX and GST (Sečenji et al., 2010; Kang et al., 2013a). In this study, drought treatment led to the upregulation of genes related to GPX, GR and APX, as well as the accumulation of glutathione and ascorbate (**Figure 9E**). This suggests that drought enhances the synthesis capacity of glutathione and ascorbate, which helps alleviate the impact of ROS on plants. The study also found that the genes involved in regulating PGD and G6PD. PGD and G6PD are involved in the conversion of NADP+ to NADPH, catalyzing the production of NADPH as a reducing agent and providing reducing power for the conversion of glutathione disulfide (GSSG) to glutathione (Noctor et al., 2012). This indicates that coenzymes providing reducing power actively respond to drought stress, in addition to the role of reductases under drought stress. Notably, most of the 28 *GST* genes were significantly upregulated under drought stress. GS is encoded by a multigene family that is primarily present in the cytoplasm. It plays a crucial role in combating oxidative stress under biotic and abiotic stresses, acting as a detoxifier for harmful substances inside and outside cells (Dixon et al., 2002). After rehydration, the gene expression and metabolites related to glutathione metabolism decreased, indicating the gradual relief of drought stress.

Amino acids play various roles in regulating plant tolerance to abiotic stress as osmoprotectants, ROS scavengers, and precursors of energy-related metabolites (Less and Galili, 2008). Serine, proline and leucine are important signaling molecules, while other amino acids are precursors of hormones and secondary metabolites with signal transduction functions (Hannah et al., 2010; Szabados and Savouré, 2010; Ros et al., 2014). You et al. (2019) found that *Sesamum indicum* accumulates a large amount of tryptophan, leucine, isoleucine, asparagine, and tyrosine under drought stress. The results of the present study demonstrate that during drought stress, the contents of l-leucine, l-isoleucine, l-serine, l-asparagine and l-tryptophan in *I. difengpi* significantly increased (**Supplementary Table S8**). Branched-chain amino acids (BCAAs) containing valine, leucine and isoleucine serve multiple functions in drought stress. On the one hand, BCAAs can serve as alternative substrates for respiratory metabolism when plants face drought stress. On the other hand, the catalytic metabolism of BCAAs can provide electrons to the respiratory electron chain under stress conditions (Xu et al., 2021). Aromatic amino acids, including phenylalanine, tyrosine, and tryptophan, can be used to synthesize secondary metabolites that have various functions in abiotic stress (Xu et al., 2019). Therefore, the increase in aromatic amino acids may enhance the accumulation of secondary metabolites. The above results suggest that the accumulation of amino acids in *I. difengpi* under drought conditions may contribute to its drought tolerance.

Polyamines, mainly putrescine, spermidine and spermine, are important growth regulators in plants. When plants are subjected to stress such as water, salinity, and low temperature, polyamines can regulate the physical and chemical properties of cell membranes, remove ROS from the body, and affect the biosynthesis of DNA, RNA, and proteins (Liu et al., 2000; Seiler and Raul, 2005). The results of the present study showed that under drought stress, genes related to putrescine and spermidine synthesis, such as ODC and SPDS, were upregulated, promoted the accumulation of putrescine and spermidine (**Figure 9B**). This indicates that *I. difengpi* mainly undergoes decarboxylation through the ornithine pathway catalyzed by ornithine decarboxylase to produce putrescine, which is then converted to spermidine as a part of the active response of the plant to drought stress (Takahashi and Kakehi, 2009).

## 5 Conclusion

As an endangered medicinal plant endemic to the karst region of China, *I. difengpi* has evolved extreme tolerance to drought stress. The present study demonstrated that *I. difengpi* actively responds to drought stress transcriptomically and metabolomically: (i) Defense systems and protective mechanisms are activated rapidly under drought stress to counteract damage. This response is characterized by altering starch and sugar metabolism and promoting photomorphogenesis. (ii) *I. difengpi* has a powerful osmoregulation mechanism, synthesizing metabolites such as d-sucrose, d-trehalose, d-glucose, glutathione, flavonoids, polyamines and amino acids, which helps alleviate the osmotic pressure on the cell membrane. In conclusion, *I. difengpi* exhibits strong drought tolerance by adopting a series of powerful defense and protection measures to delay and minimize drought-induced damage. These findings provide theoretical guidance for the ecological restoration of endemic species in karst regions.

## Data availability statement

The datasets presented in this study can be found in online repositories. The names of the repository/repositories and accession number(s) can be found below: https://www.ncbi.nlm.nih.gov/genbank/, PRJNA983054.

## Author contributions

X-JZ: Conceptualization, Investigation, Methodology, Supervision, Visualization, Writing – original draft, Writing – review & editing. CW: Conceptualization, Investigation, Methodology, Supervision, Visualization, Writing – original draft, Writing – review & editing. B-YL: Funding acquisition, Project administration, Software, Supervision, Writing – review & editing. H-LL: Investigation, Methodology, Resources, Visualization, Writing – review & editing. M-LW: Funding acquisition, Project administration, Resources, Software, Supervision, Writing – review & editing. HL: Resources, Supervision, Writing – review & editing.
