# Supplementary material for: Transcriptomic and metabolomic profiling reveals the drought tolerance mechanism of Illicium difengpi (Schisandraceae)
Source: Front Plant Sci. 2024 Jan 8;14:1284135. doi: 10.3389/fpls.2023.1284135 (PMC10800416; doi:10.3389/fpls.2023.1284135)
Supplement: Supplementary file 1 [file DataSheet_1.zip › Data Sheet 1/Data Sheet 1/Supplementary_Material.docx]

Supplementary Material

**

**

**Supplementary Figure S1. Number of upregulated and downregulated differentially expressed genes (DEGs) between two groups of *I. difengpi*. CK,** **well-watered treatment; DS, drought stress treatment; DS_R, drought**–**rehydration treatment.**

**
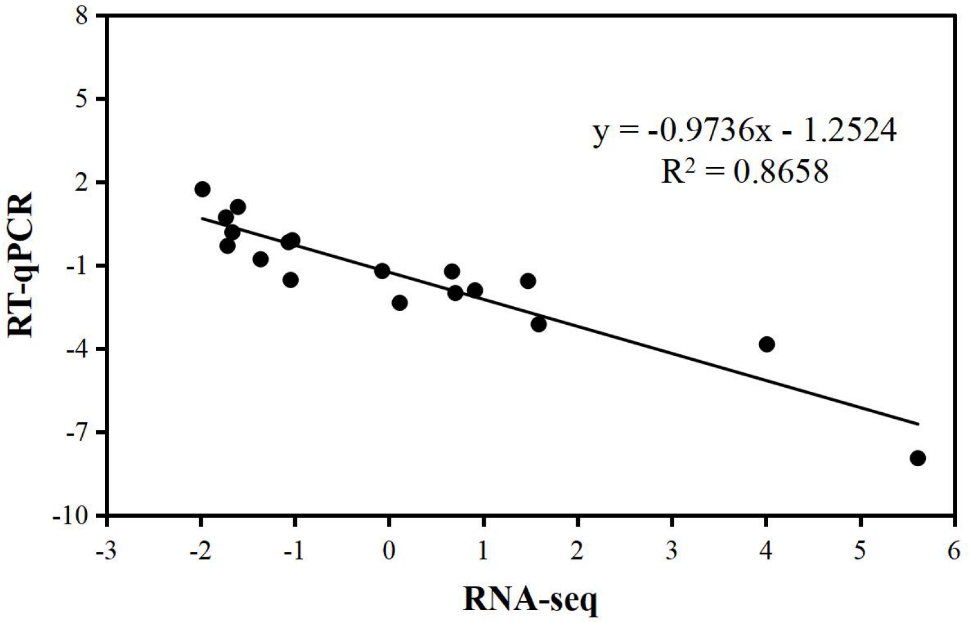
**

**Supplementary Figure S2. Correlation analysis of RT-qPCR and RNA-seq results.**

**
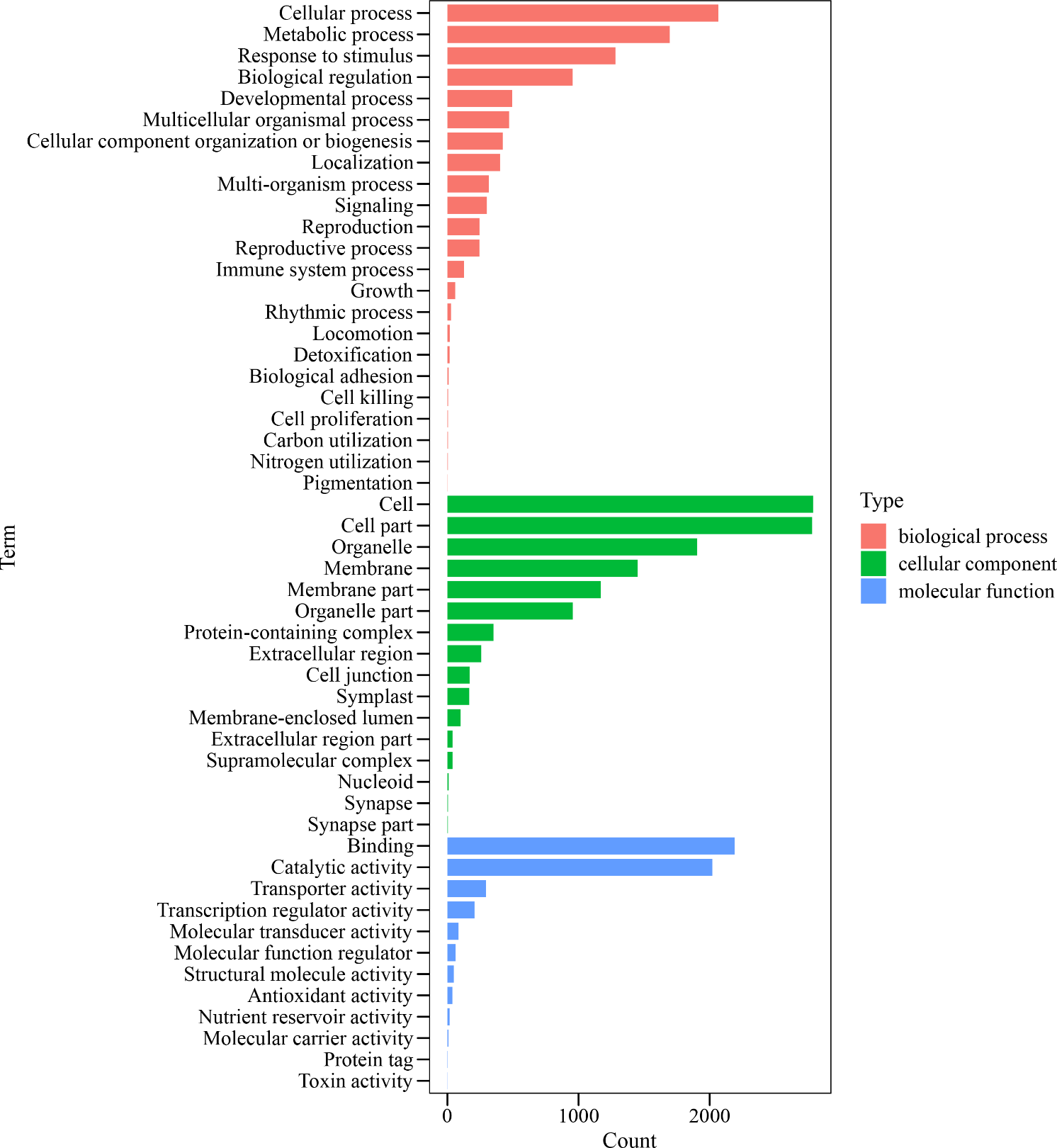
**

**A**

**
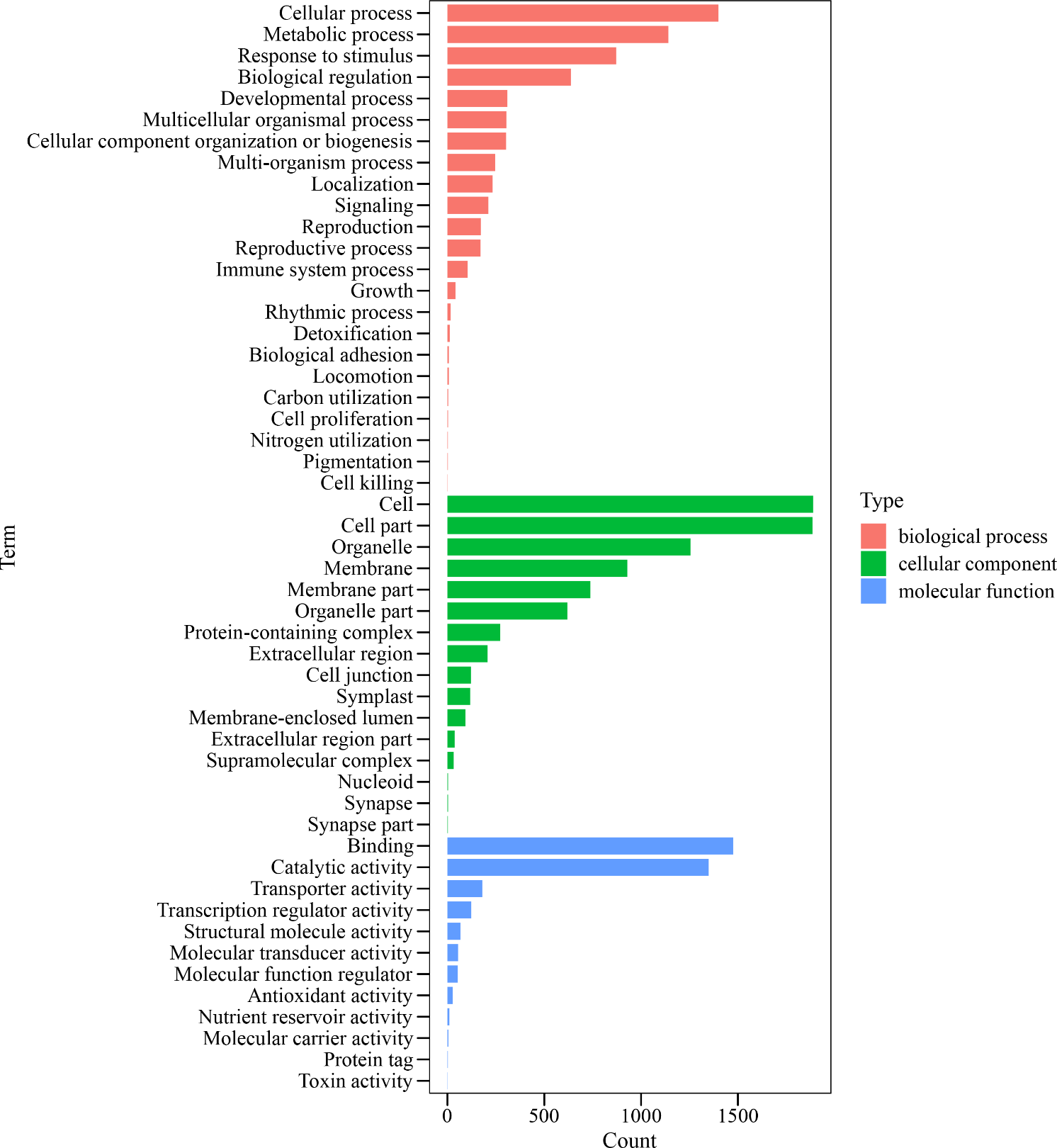
**

**B**

**
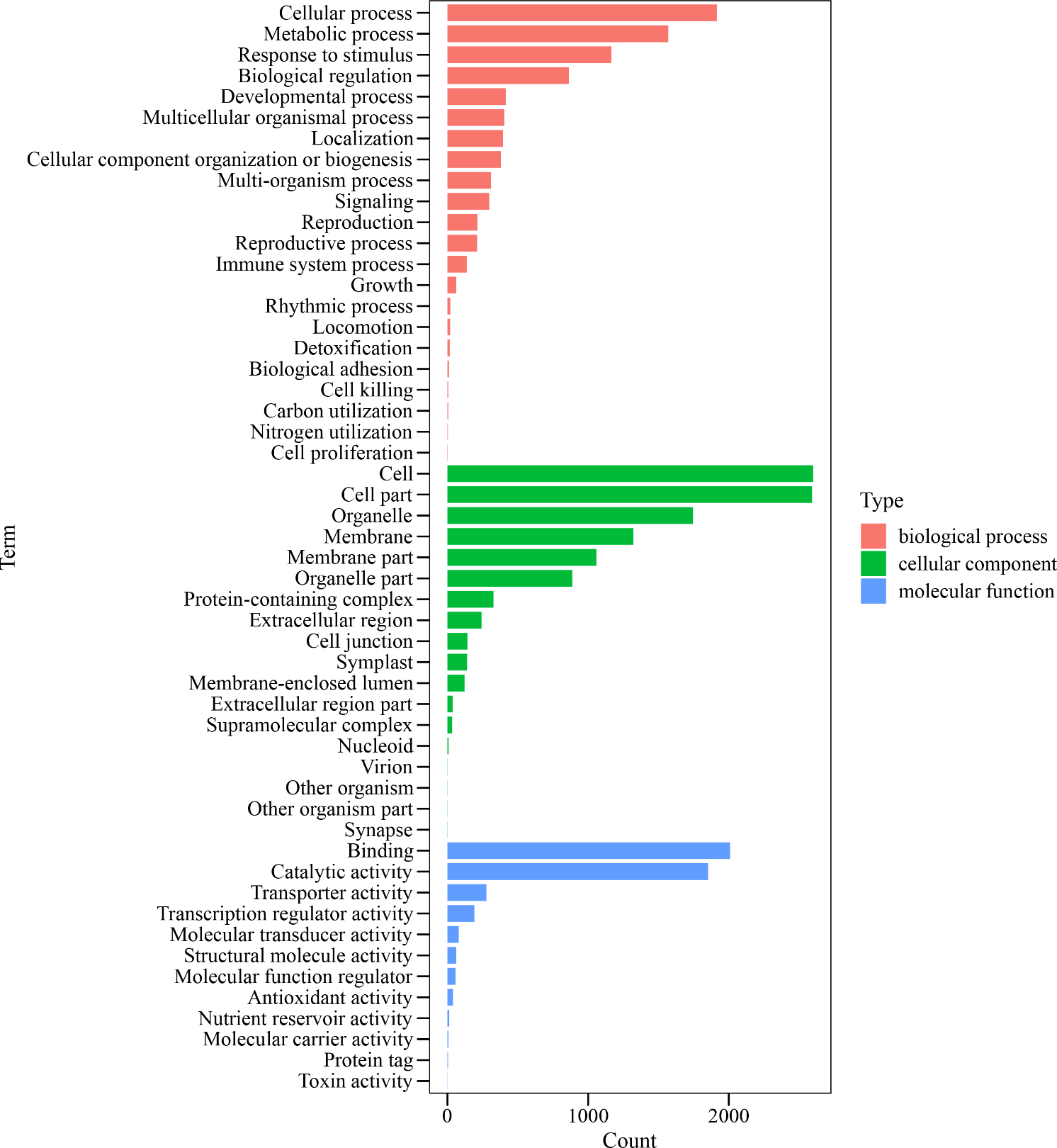
**

**C**

**Supplementary Figure S3. Gene ontology (GO) classification map of differentially expressed genes (DEGs). (A) Well-watered treatment (CK)_vs_drought stress treatment (DS). (B) Well-watered treatment (CK)_vs_drought**–**rehydration treatment (DS_R). (C) Drought stress treatment (DS)_vs_drought**–**rehydration treatment (DS_R). GO terms are listed in the abscissa, while the ordinate represents the number of DEGs tagged with the particular GO term.**

**

**

**Supplementary Figure S4. Types and proportions of the metabolites identified from *I. difengpi*.**

**

**

**Supplementary Figure S5. Number of upregulated and downregulated differential metabolites (DEMs) between two groups of *I. difengpi*. CK, well-watered treatment; DS, drought stress treatment; DS_R, drought**–**rehydration treatment.**

**A**

**

**

**

**

**B**

**

**

**C**

**Supplementary Figure S6. Types and proportions of the metabolites identified between two groups of *Illicium difengpi*. (A) Well-watered treatment (CK)_vs_drought stress treatment (DS). (B) Well-watered treatment (CK)_vs_drought**–**rehydration treatment (DS_R). (C) Drought stress treatment (DS)_vs_drought**–**rehydration treatment (DS_R).**

**

**

**A**

**

**

**B**

**

**

**C**

**Supplementary Figure S7. DEGs and DEMs KEGG enrichment pathways. (A) Well-watered treatment (CK)_vs_drought stress treatment (DS). (B) Well-watered treatment (CK)_vs_drought**–**rehydration treatment (DS_R). (C) Drought stress treatment (DS)_vs_drought**–**rehydration treatment (DS_R).**

**Supplementary Table S1. Validation of RNA-Seq data by RT-qPCR analysis of 6 selected transcripts**

| **Gene ID** | **Gene Code** | **RT-qPCR Primers (5'-3')-Forward** | **RT-qPCR Primers (5'-3')-Reverse** | **product size** |
| --- | --- | --- | --- | --- |
| *g9144_i0* | F3H | AGTTTGCTTGCCCAGTTTGC | CGTCCTCGTCAAAACCGAGA | 128 bp |
| *g1753_i0* | TPS | GGTTGTCCGGGTTACGATCC | ACCGGAAACCACACCAAGAG | 195 bp |
| *g17611_i0* | GST | GTCCACATGCACAACACGAG | GGGTTGAGAGCGAGATGAGG | 120 bp |
| *g6358_i0* | NCED3 | CGAACCGGCTAGTCCAAGAG | GCCGGTCGTTGAAGTAGACA | 186 bp |
| *g7873_i0* | SNRK2 | CCTGCCCCTCGTCTGAAAAT | ACGACCATACATCCGCCATC | 154 bp |
| *g8008_i0* | WRKY | TGGCAAGTGTGGTTCTACTGG | CCTTGGATGGGGAGATCCTT | 172 bp |
| *g11352_i0* | Actin | AGACGCAGGATAGCATGTGG | TTTCCCTTTATGCCAGCGGT | 115 bp |

**Supplementary Table S2. RNA sequencing output statistics**

| **Sample** | **Raw Reads** | **Clean Reads** | **Clean Bases(G)** | **Error Rate(%)** | **Q20(%)** | **Q30(%)** | **GC Content(%)** | **Reads mapped in single read** | **Reads not mapped** | **Mapped reads (%)** | **Unique Mapped reads** | **Secondary Mapped reads** |
| --- | --- | --- | --- | --- | --- | --- | --- | --- | --- | --- | --- | --- |
| CK1 | 49,376,632 | 4,744,1790 | 7.03 | 0.02 | 97.63 | 93.54 | 46.55 | 38,543,247 | 16381,349 | 70.17 | 32,995,283 | 5,547,964 |
| CK2 | 50,257,540 | 49,267,564 | 7.28 | 0.02 | 97.71 | 93.64 | 46.82 | 39,323,717 | 16,407,417 | 70.56 | 33,850,123 | 5,473,594 |
| CK3 | 46,430,928 | 44,008,226 | 6.50 | 0.02 | 97.53 | 93.3 | 46.53 | 35,453,984 | 16,089,317 | 68.78 | 30,341,611 | 5,112,373 |
| CK4 | 44,527,780 | 42,653,706 | 6.35 | 0.02 | 97.6 | 93.47 | 46.57 | 35,201,022 | 14,061,649 | 71.46 | 30,466,131 | 4,734,891 |
| DS1 | 55,720,644 | 52,936,194 | 7.78 | 0.02 | 97.43 | 93.15 | 46.36 | 43,804,890 | 19,140,259 | 69.59 | 36,580,385 | 7,224,505 |
| DS2 | 41,567,414 | 39,926,772 | 5.94 | 0.02 | 97.55 | 93.32 | 46.50 | 34,357,102 | 12,854,692 | 72.77 | 28,712,722 | 5,644,380 |
| DS3 | 47,938,686 | 45,769,386 | 6.69 | 0.02 | 97.72 | 93.85 | 46.89 | 38,447,090 | 16,246,443 | 70.3 | 31,692,243 | 6,754,847 |
| DS4 | 45,425,944 | 43,565,936 | 6.43 | 0.02 | 97.39 | 93.01 | 46.94 | 37,413,954 | 14,461,668 | 72.12 | 30,964,276 | 6,449,678 |
| DS_R1 | 41,816,622 | 40,234,958 | 5.99 | 0.02 | 97.43 | 93.07 | 46.24 | 33,208,443 | 12,832,907 | 72.13 | 28,983,715 | 4,224,728 |
| DS_R2 | 41,347,254 | 39,359,504 | 5.86 | 0.02 | 97.39 | 93.01 | 46.52 | 33,141,040 | 12,622,545 | 72.42 | 28,724,709 | 4,416,331 |
| DS_R3 | 43,260,170 | 41,365,842 | 6.11 | 0.02 | 97.62 | 93.53 | 46.65 | 33,419,011 | 143,320,20 | 70 | 28,938,150 | 4,480,861 |
| DS_R4 | 43,571,570 | 41,827,512 | 6.24 | 0.02 | 97.45 | 93.12 | 46.50 | 35,095,775 | 13,0580,13 | 72.88 | 30,513,557 | 4,582,218 |

Note：CK1,2,3,4,are well-watered control samples, DS1,2,3,4 are drought stress treatment samples, DS_R1,2,3,4 are drought–rehydration treatment samples.

**Supplementary Table S3. Assembly statistics**

|  | **Assembly** | **Transcript** |
| --- | --- | --- |
| N50 | 712 bp | 1,255 bp |
| Minimum | 79 bp | 79 bp |
| Maximum | 21,949 bp | 21,949 bp |
| Average | 552.63 bp | 689.19 bp |
| Count | 20,8278 | 231,784 |
| Total | 115,101,008 | 159,742,826 |
| Reads re-mapped | 71.09% |  |

**Supplementary Table S4. Validation of RNA-Seq data by RT-qPCR analysis of 6 selected transcripts**

| **Gene code** | **log2FC RT-qPCR** | **log2FC RPKM** |  |
| --- | --- | --- | --- |
| F3H | -1.98 | 1.73 | CK/DS |
| TPS | -1.36 | -0.79 |  |
| GST | -1.71 | -0.30 |  |
| NCED3 | -1.60 | 1.10 |  |
| SNRK2 | -1.73 | 0.72 |  |
| WRKY | -1.66 | 0.18 |  |
| F3H | -1.07 | -0.18 | CK/DS_R |
| TPS | 0.11 | -2.35 |  |
| GST | -1.04 | -1.53 |  |
| NCED3 | 4.01 | -3.85 |  |
| SNRK2 | -1.02 | -0.10 |  |
| WRKY | -0.07 | -1.21 |  |
| F3H | 0.91 | -1.91 | DS/DS_R |
| TPS | 1.48 | -1.57 |  |
| GST | 0.67 | -1.22 |  |
| NCED3 | 5.61 | -7.94 |  |
| SNRK2 | 0.70 | -2.00 |  |
